# Qualitation and Quantitation on Microplasma Jet for Bacteria Inactivation

**DOI:** 10.1038/srep18838

**Published:** 2016-01-06

**Authors:** ChangMing Du, Ya Liu, YaNi Huang, ZiMing Li, Rui Men, Yue Men, Jun Tang

**Affiliations:** 1 Guangdong Provincial Key Laboratory of Environmental Pollution Control and Remediation Technology, School of Environmental Science and Engineering, Sun Yat-Sen University, Guangzhou 510275, China; 2 Guangdong Experimental High School, GuangZhou, 510375, China

## Abstract

In this work, a self-made microplasma jet system was used to conduct the qualitation and quantitation of inactivation with *Escherichia coli* as the target bacteria. The logarithmic concentration and the size of antimicrobial rings served as the evaluation parameters, respectively. The effect of various parameters on inactivation effect was studied. The results showed that the majority of bacteria had been inactivated in 30 s. The inactivation effect enhanced and then weakened with the increase of air flow rate, and receded as the extension of treatment distance. The effect with different carrier gases showed as follows: oxygen > air > nitrogen > argon. Meanwhile, the effect of different components of microplasma was studied in the optimum conditions (The flow rate was 5 L/min; inactivation distance was 2 cm). The results showed that electrically neutral active species was the main factor of inactivation rather than heating effect, ultraviolet radiation and charged particles. Finally the experiments of thallus change proved that microplasma jet had etching effect on cell membrane. It also found that microplasma could degrade organic material like protein. Furthermore, the images of scanning electron microscope (SEM) revealed the change of cell morphology step by step in the whole process of inactivation.

With the development of society, people pay more attention to the control of biological pollution, which leads to the rapid development of disinfection technology. At present, disinfection technologies have been throughout various aspects of society, such as environmental field, medical field, food and tableware disinfection in everyday life and so on. Some disinfection technologies like chemicals, high temperature, high energy electron beam, X-rays, and gamma ray radiation system have been commercialized. Nevertheless, the application of those systems will not solve all the problems at present on account of cost, efficiency, power consumption, toxic residue, personnel safety and so on^1^,^2^. Thus, a variety of new relevant technologies appear. In the emerged technologies, non-thermal plasma inactivation technology shows great application prospect due to the advantages of high efficiency, wide range of adaptation, no toxic residue, the effective control of gaseous by-products, safety, low cost, etc.

The temperature of non-thermal plasma is similar to room temperature, and there are kinds of active species (free radicals, reactive atoms, reactive molecules, etc.) in plasma. Such kinds of active species are unable to obtain from the general chemical reactions. Those strengths are the key to the rapid development of non-thermal plasma.

From 1980s, non-thermal plasma started to flourish in surface modification^3^, environmental governance^4^,^5^,^6^,^7^,^8^, clean energy^9^,^10^, biomedicine^11^,^12^,^13^ and many other fields. So the research direction of multi-disciplinary cross has formed^14^,^15^. Especially in the field of biomedicine, non-thermal plasma has made encouraging achievements, and is developing continuously.

Microplasma is of small size and generated from minimum breakdown-voltage under atmospheric pressure based on the theory of Paschen’s law. The gap between electrodes is generally micron-level, which is smaller than that between the conventional plasma source electrodes^16^. The form of microplasma jet can realize accurate positioning and fine operation, which is a promising form of plasma in the field of microplasma.

Microplasma jet, as a new generation of non-thermal plasma inactivation technology, has the characteristics of low power consumption, high density, and high stability, etc. In addition, microplasma generator is smaller, more economical and more portable compared with other sterilizers. Thus, the technology arouses concern of the researchers at home and abroad.

Changming Du *et al.*^6^,^7^ have proved that the *Escherichia coli* on the surface of materials or in water could be inactivated by non-thermal arc efficiently. Besides, they have also explored the deactivation mechanism of non-thermal on *Escherichia coli* and found that the active species in plasma was the key factor in inactivation.

In this work, a self-made microplasma jet system was used to conduct the qualitative and quantitative researches of inactivation to explore the optimum parameters. In addition, different devices of separation were used to study the effect of components in plasma on inactivation. And the main factor of inactivation was determined in microplasma.

## Materials and Methods

### Portable microplasma jet

**Figure 1:**
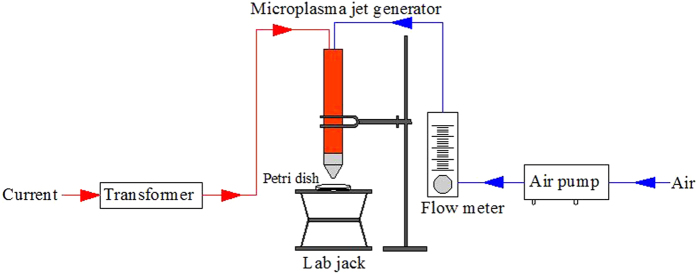
The experimental device: it is mainly composed of the power supply system, gas supply system and the microplasma generation system.

The experimental device of the work is shown as Fig. 1, which is mainly composed of the power supply system, gas supply system and the microplasma generation system. The power supply system is mainly a single-phase ac transformer and the input voltage is the standard voltage (220 V, 50 Hz). The gas supply system mainly includes air pump, gas cylinder and flow meter. They are used to provide air for microplasma generator, provide other types of carrier gases (oxygen, nitrogen and argon) and control the gas flow rate, respectively.

Microplasma jet generator, the core component of the experiment system, is composed of two copper electrodes. The inner electrode is hollow copper pipe; the outer electrode is a cylinder with a nozzle (The diameter is 1 mm.) at the top. Besides, the transformer provides ac voltage to the inner and outer electrodes, between which there is discharge. Gas ionization occurs when gas enters the discharge gap and disruptive discharge takes place. Then the ionized gas is carried out, forming the visual microplasma jet.

### Experiment methods

Standard strains of *E. coli* ATCC 25922 (Gram-negative bacteria) were used throughout the study to prepare specimens. The expanding training process of primitive bacterium solution is as follows: take a ring of *E. coli* from the slope to inoculate into the conical flask containing 50 mL LB broth, and then put it in water-bathing constant temperature vibrator (120 r/min, 37 °C) to cultivate for 14–16 hours. Next bring 10 mL bacterium solution described above to the 50 mL sterile centrifuge tube and centrifuge for 10 minutes (4000 rpm, 20 °C). Then remove the supernatant and add 10 mL sterile water to confect primitive bacterium solution.

In qualitative inactivation experiments, take 0.1 mL of the primitive bacterium solution and coat on the prepared plate evenly. Then conduct the microplasma inactivation treatment once the bacterium solution is absorbed completely. Eventually put the treated plates in the incubator of 37 °C for 24 hours.

In quantitative inactivation experiments, cut the mixed cellulose ester membrane filte (diameter: 0.5 mm; aperture: 0.22 μm) into membrane filter pieces of 1 × 1 cm. Then put the velum block to dry and sterile culture dishes, and open them under the UV lamps to sterilize for 5 minutes. Next take 10 μL (bacterial counts: 10^7^–10^9^) of the primitive bacterium solution on the membrane filter block. Then conduct the microplasma inactivation treatment once the bacterium solution is absorbed completely. Put the membrane filter block into the 50 mL screw centrifuge tube containing 10 mL sterile water immediately after the treatment and then conduct dilution plating procedure. Each plate is inoculated 100 μL bacteria solution. Eventually put the treated plates in the incubator of 37 °C for 24 hours.

Quantitative experimental method is convenient, quick and easy to operate, but the volume of bacterium solution is only 10 μL, which limits the experiments of mechanism. So the experimental method of mechanism is improved. Draw square grids of 1 × 1 cm on the mixed cellulose ester membrane filte (diameter: 47 mm; aperture: 0.22 μm). Then cover the membrane on the filter and take 5 mL sterile water to clean it. Next connect to vacuum pump until water disappears. Add 10 mL of the primitive bacteria solution when the pressure becomes zero, and then conduct the suction filtration until the membrane surface is completely dry. The whole process takes no more than 20 s. Cut the membrane into a 1 × 1 cm velum along with the grids in sterile conditions. Then treat the velum blocks subpackaged in the sterile and dry plates in different conditions. Put the membrane filter block into the 50 mL screw centrifuge tube containing 10 mL sterile water immediately after treatment and then conduct dilution plating procedure.

Count the bacterial colonies after training for 24 hours. The counting results are expressed in bacteria concentration, namely plate colony number multiplied by the dilution ratio, and inactivation effect mainly shows as the logarithmic reduction value and the inactivation rate. The specific formula is as follows:


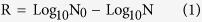



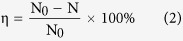


Where R is logarithmic reduction value; n is bacteria inactivation rate; N_0_ and N respectively represent the bacterial concentration before and after the plasma treatment (unit: CFU/mL).

## Methods of mechanism analysis

### Inductively coupled plasma atomic emission spectrometry (ICP-AES)

ICP-AES is a spectral analysis method which takes inductively coupled plasma moment as the excitation light source. The method possesses the advantages of high accuracy and low limitation of detection. It has the capability to determinate multi-element simultaneously. In the experiments, ICP-AES was used to detect the content of K^+^ and Mg^2+^ in bacteria solution after treatment, which can characterize the permeability change of cell membrane of the treated bacterial. Test conditions are as follows: incident power = 1300 W; plasma gas flow = 12 L/min; atomizing gas flow = 0.55 L/min; detection is based on JY–T 015-1996 (general principles of ICP-AES).

### Three-dimensiona excitation-emission matrix spectroscopy (3D-EEM)

3D-EEM possesses high sensitivity and selectivity, which can be used for classification and quantitative determination of dissolving organic matter in water. The detection principle is as follows: the soluble organic matter in water is exposed to light irradiation with specific wavelength from 3D-EEM (Aqualog-UV-800, France), and the electron will be excited to jump. But due to that state is unstable and electron will eventually return to the ground state. Thus the energy emits in the form of fluorescence. And then 3D-EEM is obtained through the intensity of the excitation and emission.

### Cold Field Emission Scanning Electron Microscope (SEM)

SEM can be used to observe the images of secondary electron and scattering electron, which can be applied to the surface observation of morphology in the fields of biology, physics, chemistry, material and so on. The most obvious advantages of cold field emission are the relatively small diameter of electron beam, high brightness and high resolution. The research adopts SEM (JSM-6330F, Japan) to characterize exterior morphology of the bacteria so as to analyze the etching effect of microplasma on bacterial cells.

## Results and Discussion

### Basic researches of microplasma jet inactivation

#### Volt-ampere characteristic

Volt-ampere characteristic is one of the most important electrical parameter characteristics of microplasma jet generator. It has to do with gas flow rate, gas type, voltage and electrode distance, etc^17^. Through the analysis of current and voltage change, the ionization characteristics of microplasma and energy consumption can be understood more clearly. And that is related to the inactivation efficiency and application prospect of the device.

**Figure 2:**
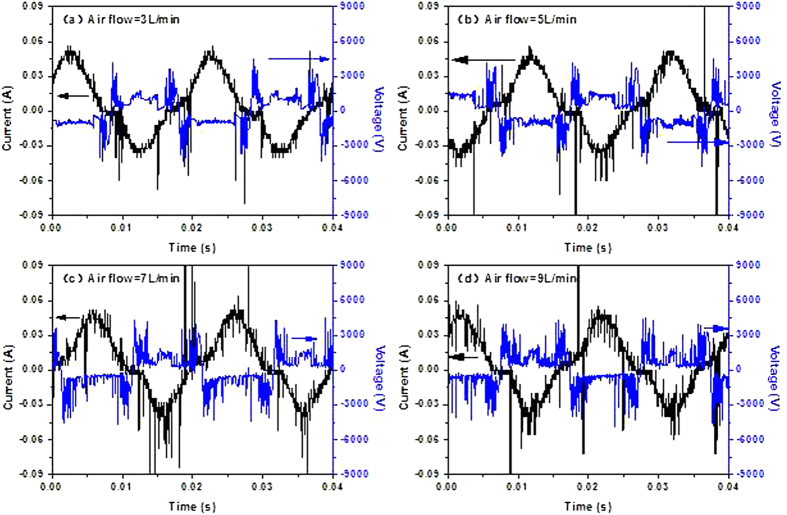
Volt-ampere characteristics with different air flow (3 L/min, 5 L/min, 7 L/min, 9 L/min): the serrated fluctuation increases significantly with increasing air flow.

In the experiments, tektronix oscilloscope, high pressure probe and current probe were used to measure the current and voltage change of the plasma generator. Volt-ampere characteristics with different air flow were studied in order to explore the influence. The results are shown in Fig. 2.

As can be seen from Fig. 2, the microplasma jet discharge is unstable, but the voltage and current cyclically change and the cycle frequency is 50 Hz. The current changes with sinusoidal characteristics, and there are two current pulses in a voltage cycle. And positive current pulse value is greater than the negative current pulses.

In Fig. 2a–d, the air flows are 3 L/min, 5 L/min, 7 L/min and 9 L/min, respectively. It can be found that the serrated fluctuation increases significantly with increasing air flow through the comparison. It proves that improving air flow will lead to an unstable discharge. It may be due to that the effect of the turbulence of high-speed air flow leads to the frequent occurrence of the suspension of discharge and once more breakdown. Thus it can be seen breakdown occurs more frequently at high air flow, which is beneficial to the generation of active species through air ionization. But the bigger is not the better. Because the growth rate of ionization producing active species cannot keep up with the increase of gas flow. So the unit flow rate of the active species reduces actually. What is more, high velocity causes that airflow containing active species goes through the target area quickly. That may weaken the efficiency as well. Therefore, the suitable flow needs to be founded to ensure enough generation of active species so as to realize satisfactory inactivation effect.

Through computing the integral for the data of current and voltage within 0.1 s, the discharge power can be obtained under different air flow rates (3 L/min, 5 L/min, 7 L/min, 9 L/min). Those are 21.57 W 23.03 W, 18.61 W and 16.58 W, respectively. The results show that the discharge power increases and then decreases with the augment of flow, and it reaches the maximum at the flow rate of 5 L/min. Uhm, H. S. *et al.*^18^ have studied the inactivation effect of radio frequency plasma jet device with different discharge power and found that with the increase of operating power, inactivation quantity increased as well. In the following study it found that the optimum air flow rate is 5 L/min, which is chosen in the follow-up experiments.

**Figure 3:**
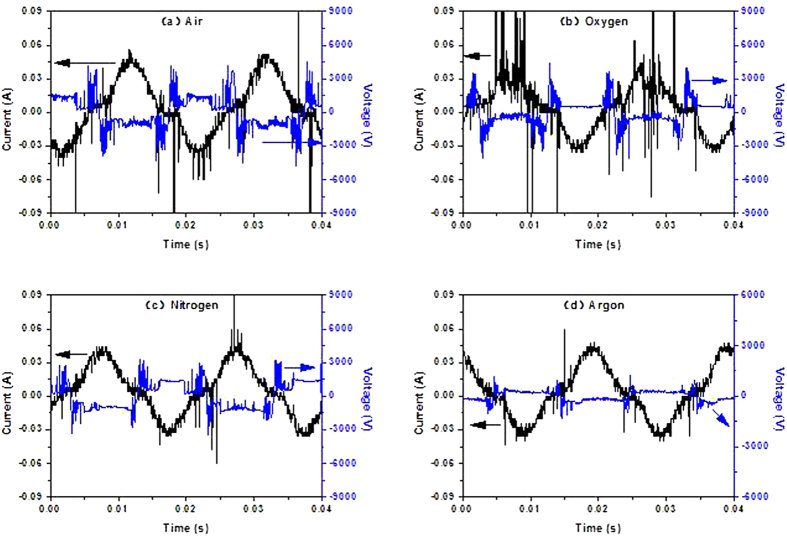
The diagrams of volt-ampere characteristics with air, oxygen, nitrogen and argon gas as carries gases: the current of oxygen microplasma fluctuates most intensely; the smallest is that of argon plasma.

The state of microplasma ionization also has relationship with gas type. Different types of gas lead to different degrees of ionization. And it will affect the inactivation effect. So the diagrams of volt-ampere characteristics were measured with air, oxygen, nitrogen and argon gas as carries gases at the flow rate of 5 L/min (as shown in Fig. 3).

As can be seen from Fig. 3, the current of oxygen microplasma fluctuates most intensely in the same condition. It indicates that the frequency of gas breakdown is the highest. Surely the degree of ionization is the highest at the same flow rate. And air ionization degree is slightly higher than that of nitrogen. The worst is argon plasma among air, nitrogen and argon plasma and the range of voltage and current variation is the narrowest, correspondingly the energy consumption is the smallest. Different ionization degree mainly results from different bond energy. Some studies have showed that the order of bond energy is as follows: oxygen < nitrogen < argon, and nitrogen and oxygen is the most abundant in the air, so its ionization degree is in between^19^,^20^. The difference of ionization degree decides the inactivation efficiency of various kinds of plasma. It will be discussed in the subsequent paper.

### Effect of air flow

**Figure 4:**
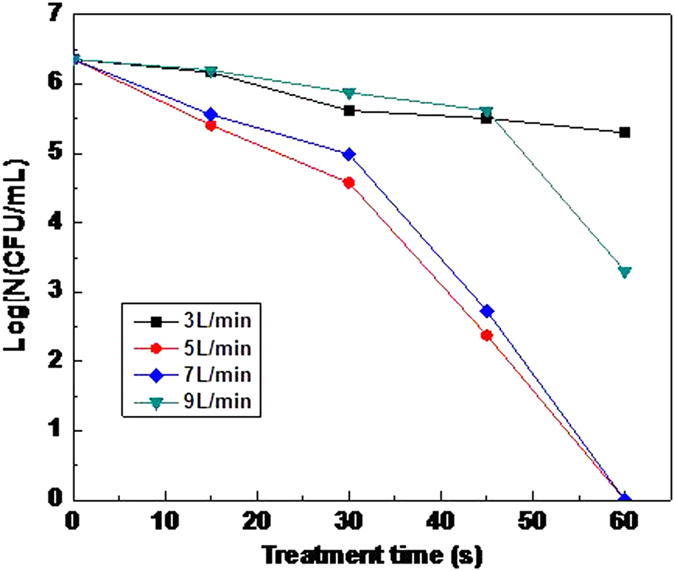
The inactivation effect of different treatment time with various air flow rates: that of air flow of 5 L/min is the best, and that of air flow of 7 L/min is a bit worse; the inactivation effect with air flow of 9 L/min is the worst.

It can be known that air flow influence the stability of discharge from the analysis of volt-ampere characteristic. So it is necessary to explore the effect of air flow on microplasma jet inactivation. In the experiments, the inactivation distance was set to 2 cm, and the research of single factor was conducted with the air flow as a variable. The inactivation effect of different treatment time with various air flow rates is shown in Fig. 4.

The experimental results show that the inactivation effect with air flow of 5 L/min is the best, and 7 L/min is a bit worse. Nevertheless both can make the bacteria inactivated completely within 60 s, and the inactivation rate is similar. While the inactivation effect with air flow of 9 L/min is far away from the former two, and the logarithm reduction of bacteria is only 3.06 within 60 s. It suggests that the greater air flow rate does not mean the better inactivation effect. And it can be concluded that greater flow will leave more air ionized, thus the ionization material increases accordingly. However, inactivation effect is worse conversely. That is mainly because the increment of air ionization matter caused by the increase of flow cause is far behind the pace of the increase of air. It results in that the active species in the unit of flow in the microplasma jet does not rise in fact. Besides, high flow rate under high velocity leads to that a lot of active species has no enough time to act on bacterial but has be taken away.

The logarithm reduction of bacteria is only 1.06 within 60 s with the air flow of 3 L/min, which shows that too small flow cannot improve the efficiency of inactivation as well. That is because the airflow is not able to blow the active species generated in the discharge area to the target from the nozzle in time. So the appropriate air flow is an important guarantee to achieve the ideal effect. Gas flow of 5 L/min is selected in the subsequent experiments.

### Effect of distance

**Figure 5:**
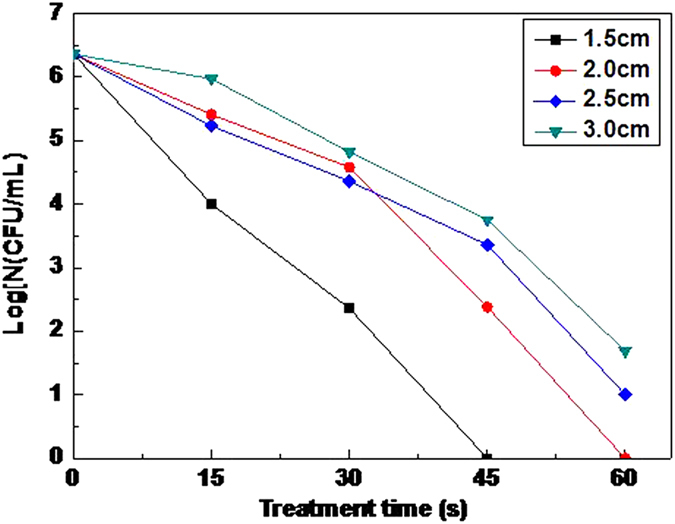
The effect of inactivation distance: Inactivation effect becomes poorer with the increase of inactivation distance.

Inactivation distance refers to the distance between the microplasma generator nozzle and the target. In the experiments, the air flow rate was set at 5 L/min, and the distance was made as to the single factor variable to study the inactivation effect. The result is shown in Fig. 5.

The experimental results show that with the increase of the distance, inactivation effect is poorer. The best is the distance of 1.5 cm, in which condition the bacteria can almost be inactivated completely within 45 s. Besides, the distance of 2 cm can realize that in 60 s as well. Compared with the logarithm reduction of 6.36, the distance of 2.5 cm can achieve to 5.36 in 60 s. And 3.0 cm is poorer, of which is only 4.67. Varieties of active particles generated from plasma ionization are the key of inactivation. But many of the active particles have no long lifetime in the environment. They are easy to decay or react with other components. Some active particles only have a life of millisecond and even microsecond^21^. And the longer treatment distance causes the longer time from the generation of active particles to working on the bacteria. The lower concentration of active particles, the smaller the etching effect on bacteria is. What is more, shorter-lived active substance has higher inactivation effect than the longevity^22^. So it appeared that with the increase of distance, the inactivation efficiency weakened. But the shorter is not the better. It is due to that the shorter distance means the stronger heating effect, which is much adverse to the temperature sensitive material and the operating personnel.

### Effect of carrier gas type

**Figure 6:**
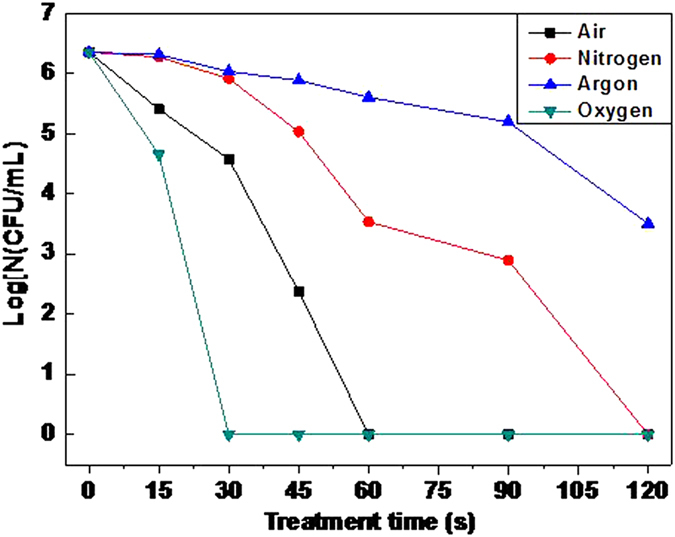
The inactivation effect of microplasma jet with different types of carrier gases: the order of inactivation ability of the four gas microplasma is as follows: oxygen > air > nitrogen >argon.

Inactivation effect of microplasma jet was studied with air, oxygen, nitrogen and argon as carrier gases with the flow of 5 L/min and inactivation distance of 2 cm. The result is shown in Fig. 6.

According to the results, the order of inactivation ability of the four types of gas microplasma is as follows: oxygen > air > nitrogen >argon. The oxygen plasma could inactivate almost all the bacteria within 30 s, while air plasma needed 60 s, and nitrogen needed 120 s. The worst was the argon plasma, of which there still remained a lot of survived bacteria even in two minutes. Compared to the volt-ampere characteristic of different gas in Fig. 3, the following conclusions can be drawn.

In these gas molecules, molecular oxygen is most easily ionized. It is due to that oxygen plasma can produce a large number of oxidizing substances. And compared with active species produced by other gases, reactive oxygen species produced by oxygen is more competitive in inactivating bacteria. So the inactivation ability of oxygen microplasma is much higher than air plasma. Current research has affirmed that in the substances of plasma, oxygen ionization accounts for a large part, mainly including atomic oxygen (O)^23^,^24^,^25^,^26^,^27^, ozone (O_3_)^24^, metastable O_2_^24^ and oxygen anion (O^2−^)^28^.

Air composition is complicated, containing nitrogen, oxygen, inert gas, water vapor and so on. So the ionization process is complicated, which leads to the formed compositions of active species are relatively more. The main active species includes short-life radicals like OH•, NO• and O•, and longevity reactive molecules like ozone and hydrogen peroxide^21^. The collection of active species equips air plasma with strong inactivation ability.

The inactivation effect of nitrogen microplasma is much less than oxygen and air. It results from that the nitrogen-nitrogen triple bond of nitrogen molecules is very stable and difficult to ionize^19^. Therefore it is difficult to produce enough active species relative to oxygen. Some researchers have verified that the radical of nitric oxide (NO·) is the main inactivation material of ionized nitrogen plasma with nitrogen as carrier gas^19^.

Argon, a kind of inert gas, is extremely difficult to ionize. The results show that the inactivation effect is the poorest taking argon as carrier gas It is because argon cannot be effectively ionized to produce enough active species in the same conditions. The reason is also be that inactivation ability of a small amount of active particles produced by ionization is limited.

In conclusion, the bond energy of gas molecules determines the ionization degree in the discharge region of microplasma with the same gas flow, resulting in different concentration of active components in the microplasma jet. Furthermore, the inactivation effect of every kind of active species is different, which leads to different inactivation effect of gas microplasma. That is consistent with the results of volt-ampere characteristics in Fig. 3, which indicates that ionization degree determines the inactivation effect.

In the study, the inactivation effect of air microplasma is less than oxygen, but it can also fully meet the requirements. In addition, it is much cheaper and easier to get. So air was used as carrier gas in the follow-up experiments.

## Effect factors of microplasma jet inactivation

### Physical factors

Among the physical factors, mechanical force refers to the impact of the gas flow. But many studies have shown that the bactericidal function of the impact is quite small. Thus, it can be almost negligible and only has inactivation effect under very high speed of air flow. So in this study, the effect of mechanical force on bacteria was not considered due to that the flow velocity is relatively small.

### Heating effect

In the experiments, the infrared thermometer was used to determine the heating effect of the microplasma jet. It has found that the temperature increased rapidly within the first 30 s, and the change was not obvious after 30 s. Besides, as can be known from the previous experiments, the first 30 s was the key to inactivating, and the inactivation rate achieved to 98.4%, while the measured temperature was 28 °C at that time. Studies have shown that in most cases, heating effect can cause cell irreversible damage when the temperature is more than 43 °C^29^. That is to say, microplasma jet has realized high efficient inactivation in 30 s, while the temperature has not reached the extent of inactivating *E. coli* effectively. It suggests that heating effect is not the main cause of microplasma inactivation.

Mohamed *et al.*^30^ used air, nitrogen and oxygen as the carrier gases to study microplasma jet. And it found that the temperature of plasma airflow mainly depended on the gas flow velocity and discharge current. It has shown that high inactivation temperature could be obtained through controlling the air flow rate, inactivation distance and discharge current so as to improve the efficiency of inactivation.

### Ultraviolet radiation

**Figure 7:**
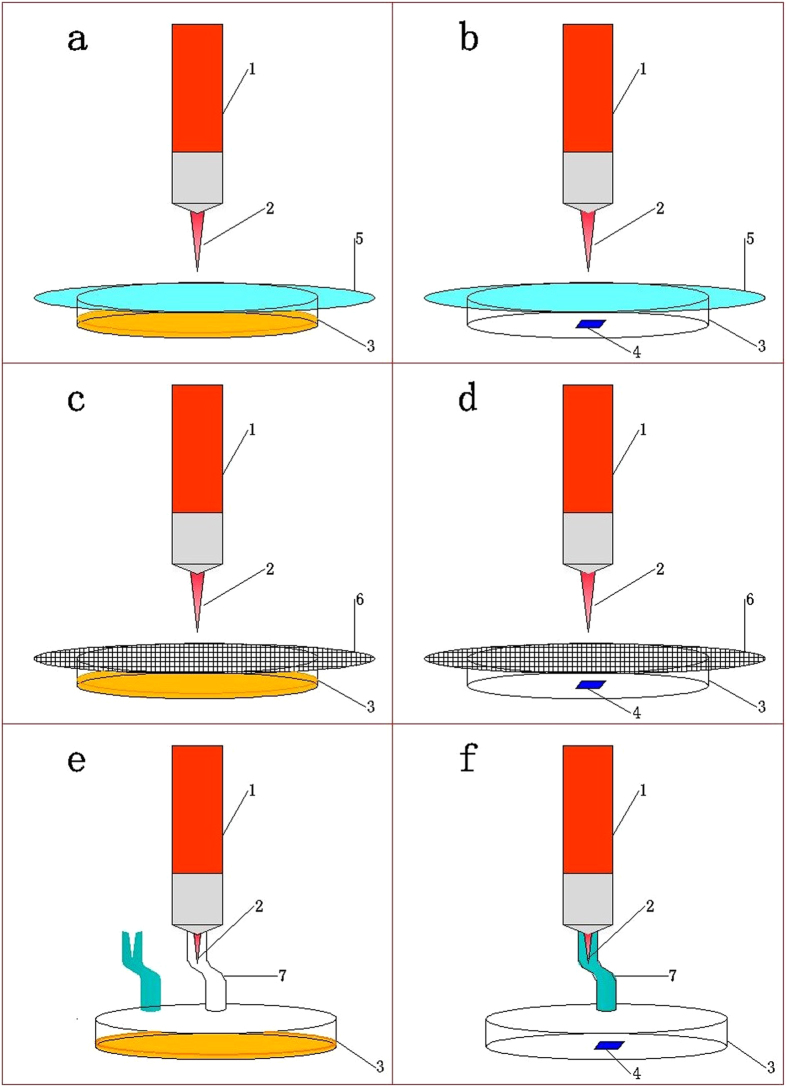
The separating devices of ultraviolet radiation (a,b), charged particles (c,d) and active species (e,f): a, c and e were used in qualitative; b, d and f were used in quantitative research. (1 - The body of the plasma generator, 2 - Microplasma jet, 3 - Petri dish, 4 - The sample, 5 - Quartz glass, 6 - The metal mesh screen, 7 - Nylon tube)

**Figure 8:**
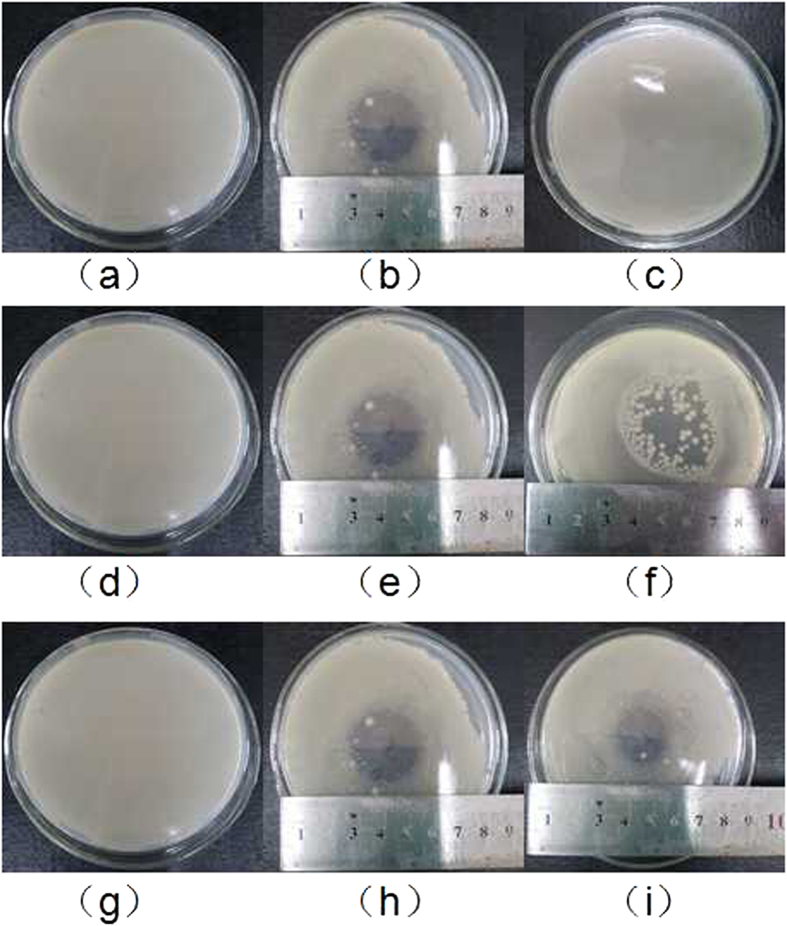
The qualitative analysis of ultraviolet radiation, charged particles and active species (a, d, g- untreated, b, e, h - treated with microplasma for 120 s, c - treated with ultraviolet radiation isolated for 120 s, f - c-treated with microplasma rid of charged particles for 120 s, i - treated with active species for 120 s)

**Figure 9:**
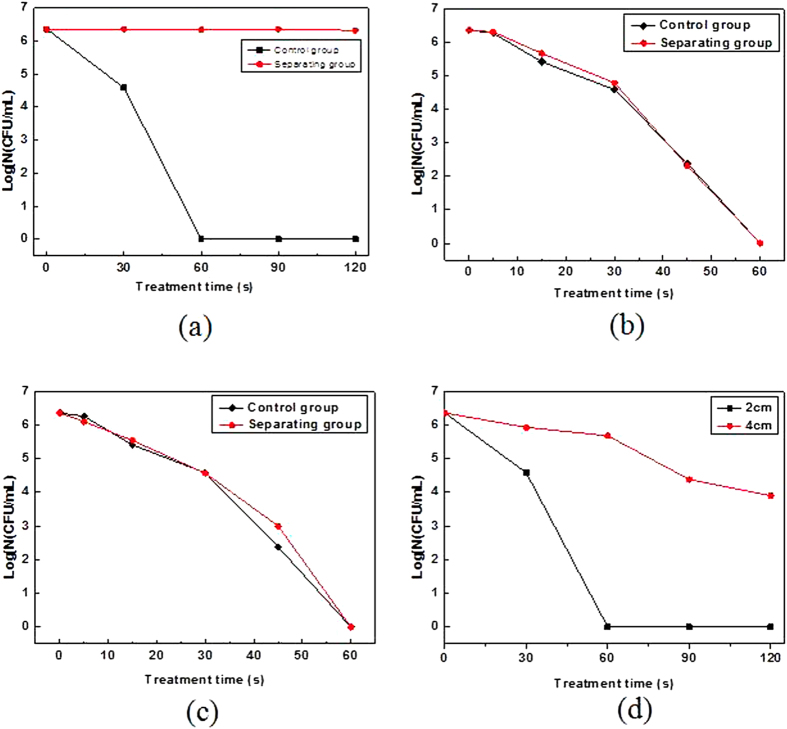
The quantitative analysis of ultraviolet radiation (**a**), charged particles (**b**), active species (**c**) and the inactivation effect with different length of ducts (**d**). (**a**) The logarithmic reduction of control group remains basically the same; (**b**) Inactivation effect of the separation group are almost the same as the control one; (**c**) There is not big difference between the control and separating group within 30 minutes before, and the bacteria of both can almost be inactivated completely in 60 s; (**d**) Longer spreading distance of plasma leads to worse inactivation effect.

Separating experiments were conducted to explore the effect of ultraviolet radiation in microplasma jet on inactivation. In the experiments, put the circular quartz glass (diameter: 10 cm; thickness: 1.5 mm) onto the culture dish (diameter: 9 cm; height: 1.7 cm) to let ultraviolet radiation in but block other microplasma components. The device is as shown in Fig. 7, of which Fig. 7a,b are qualitative and quantitative research, respectively. The changes of antimicrobial rings and the logarithm reduction of concentration are shown in Fig. 8(a–c) and Fig. 9(a) before and after the separation.

As can be seen from Fig. 8(c), there is not antimicrobial ring after the ultraviolet radiation treatment for 120 s as the untreated one. But an obvious antimicrobial ring appeared on the agar plate directly exposed to the microplasma. In Fig. 9(a), the logarithmic reduction of control group remains basically the same, the change of which is not obvious compared with the untreated. The results of qualitative and quantitative analysis show that ultraviolet radiation generated by the microplasma jet almost has no function of inactivation.

Many teams have studied the effect of ultraviolet radiation in plasma on inactivation. Boudam etc.^31^ have proved that ultraviolet radiation plays a major role in the process of plasma inactivation in certain conditions. But the carrier gas is very special, which is composed of a large number of N_2_ and small amounts of oxidizing gas (N_2_O). And it was also found that the effect of ultraviolet radiation disappeared in the discharge with air as carrier gas.

### Charged particles

Separating experiments were conducted to explore the effect of charged particles in microplasma jet on inactivation in the way of qualitative and quantitative measurement. In the experiments, put the metal mesh screen with good conductivity (500 mesh) onto the culture dish (diameter: 9 cm; height: 1.7 cm) to filter out charged particles in microplasma. The device is as shown in Fig. 7 (c - qualitative analysis; d - quantitative analysis).

The changes of antimicrobial rings and the results of logarithm reduction of concentration are as shown in Fig. 8(d–f) and 9(b) before and after the separation. What calls for special attention is that the result is not the inactivation effect of charged particles but that of the removal of charged particles.

There is no big difference between the antimicrobial rings of the unseparated and separated treatment from Fig. 8. But some colonies appear in the latter ring (as shown in Fig. 8(f), which shows that inactivation effect of the latter was slightly inferior to the former. As can be seen in Fig. 9(b), inactivation effect of the separation group is almost the same as the control one. And only subtle difference exists in the process of inactivation in the first 30 s. With the extension of time, the two groups both achieved the effect of inactivating almost all the bacteria within 60 s. It shows that microplasma jet still have very strong inactivation ability even though the charged particles are separated. The main effect of inactivation in the microplasma jet is neutral active species, and charged particles may play a positive role.

That is mainly related to the structure of the jet device. In the experiments, the formation area of microplasma is restricted inside the generator, and bacteria to be processed are only exposed to the outside. That is to say, plasma is not directly exposed to the bacteria. But in the process of plasma transmission, the rapid restructuring of electrons and ions leads to the low concentration of charged particles in the jet^32^. And that makes the inactivation effect not obvious.

### Chemical fators

Chemical factors mainly refer to the active species. The main active species in oxygen plasma is reactive oxygen species and that in nitrogen plasma is reactive nitrogen species. Both of them exist in air plasma. Furthermore, as a result of the existence of water vapor, there is some active species that the former two do not have. Essentially active species mainly includes oxygen radical (O·), hydroxyl radical (OH ·), superoxide anion (H_2_O_2_) and ozone (O_3_), etc^33^. The active species is produced through different reactions, such as electron impact excitation and decompose^34^. They can be divided into two types in the existence time, namely longevity and short-lived active species. Short-lived active species mainly include oxygen (O·), hydroxyl radical (OH ·), hydrogen peroxide ion (H_2_O_2_ -), etc. Longevity active species mainly include hydrogen peroxide (H_2_O_2_), ozone (O_3_), neutral molecules, etc^21^.

### Active species

It has proved in the previous experiments that the most important effect factor is the neutral active species rather than the ultraviolet radiation and charged particles in the process of microplasma inactivation. To further explore the inactivation effect of active species in microplasma jet, the qualitative and quantitative experiments were conducted. To separate the active species, nylon tube was connected to the end of the generator nozzle to ensure that the gas is not a direct object. In addition, a piece of metal mesh was covered on the culture dish to block charged particles and ultraviolet. The other side of the tube was set above the dish to ensure that the distance between the nozzle and the center of the dish is 2 cm. The separation device is shown in Fig. 7 (e - qualitative analysis; f - quantitative analysis).

Because the device removed the charged particles and ultraviolet radiation and only active species existed, also the nozzle and the catheter were sealed, the microplasma jet can be effectively prevented from reacting with the material in environment in the process of transmission. The changes of antifungal rings and bacteria quantity are shown in Figs 8(g,i) and 9(c).

In Fig. 8(i), the diameter of antifungal ring generated by the active species is slightly less than that generated by the unsegregated microplasma. It is mainly due to that the inactivation range is limited by the duct transporting the active species to the agar board. And in Fig. 9(c), there is not big difference between the inactivation effects of active species separated by the duct and sieve within 30 minutes before. Only at 45 s, there is 0.62 log CFU/mL apart between the separation group and control group. That indicates that the synergy inactivation effect of the ultraviolet radiation and charged particles in microplasma jet can be negligible, and that of active species is the main factor. In order to explore what component played main role exactly, different length of ducts were used to separate the active species, and the result is shown in Fig. 9(d).

As can be seen from Fig. 9(d), longer spreading distance of plasma leads to worse inactivation effect. Active species separated by the catheter of 2 cm can inactivate almost all the bacteria in 60 s, while that of 4 cm still remained 3.9 log CFU/mL even after 120 s.

That is because too long catheter leads to the unstable active species partly wear down before contacting with the bacteria. It means that either longevity or short-lived active species has intense inactivation effect, and that of the short-lived active species is higher relatively. Of course, more longevity active species is expected in practice, which can provide sustained inactivation effect.

**Figure 10:**
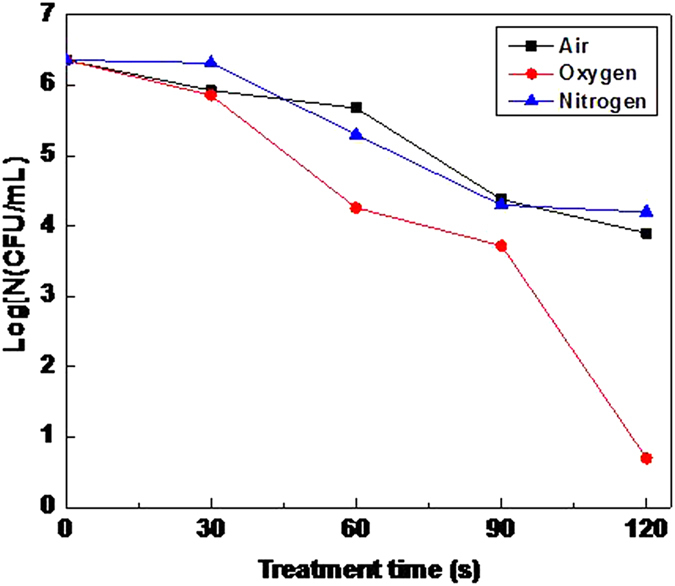
The inactivation effect of active species in microplasma of different types of gas: the inactivation effect of oxygen plasma is the best; that of air and nitrogen are approximately the same.

Due to the complexity of the composition in air microplasma, it is difficult to explore the inactivation effect of a certain component in active species. Thus, with two of the main compositions-oxygen and nitrogen in the air as carrier gases, longevity active species was made further study on the basis of separation by the duct of 4 cm. The experimental results are shown in Fig. 10.

It can be found from Fig. 10 that among the three different carrier gases, the inactivation effect of oxygen plasma is the best. While that of air and nitrogen are approximately the same. The logarithm concentration is 3.9 and 4.2, respectively. The main reasons for that case are as follows:

O_2_ and N_2_ can both disintegrate and produce radicals and charged particles under the high-energy electron bombardment in plasma discharge area. The dissociation reactions are as follows:


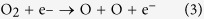



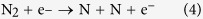


O radical possesses strong oxidizability. That equips it with the ability of reacting with other components in the plasma to generate free radicals or other reactive molecules. So the O_2_ microplasma jet has strong inactivation performance. While air contains 21% O_2_, so strong inactivation effect does it have.

The bonding energy of N ≡ N is higher (9.8 eV), nevertheless that of O = O is only 5.2 eV. So compared with O_2_, N_2_ is difficult to dissociate^19^,^35^. It results in that the concentration of active species in the microplasma jet is lower with N_2_ as carrier gas in the same condition, making worse inactivation effect than O_2_ microplasma.

### Ozone

Ozone (O_3_) is a kind of important inactivation material among the active species in air plasma. It attains the goal of inactivation through damaging the membrane structure and changing the permeability of microorganism cell by oxidation^24^. What is more, O_3_ is a kind of longevity active component. It has significant advantages of continuous inactivation. The involving reactions of O_3_ production are as follows:


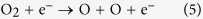



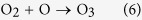


In the experiments, the change of O_3_ concentration under different air flow rates was detected with the handheld ozone detector setting below the jet. And the distance between the gas import of the instrument and the nozzle was 2 cm. It found that with the increase of air flow rate, the O_3_ concentration rised and then decreased 2 cm below the nozzle. It is consistent with the previous studies of inactivation effects with varying air flows. And both of that reach the peak at about 5 L/min. That suggests the concentration of active species represented by ozone achieves the maximum in the plasma, so as to obtain the best inactivation effect. It can also be seen that exorbitant air flow leads to intense fluctuation of O_3_ concentration. That reflects that the discharge is unstable, which is accordant with the volt-ampere characteristic diagram in Fig. 2.

Meanwhile, the ozone concentration 2 cm below the microplasma jet was measured with O_2_ and N_2_ of 5 L/min as carrier gases in the same way. It has showed that the concentration of ozone is different in microplasma jet of different types of gas. Below the nozzle of 2 cm, the concentration of ozone generated by oxygen is 108 ± 4 ppm, which is much higher than air and nitrogen. And that of air microplasma is slightly higher than N_2_, of which the wave range is 60.5 ± 1.5 ppm. It is the same with what reflects in Fig. 9(d), which indicates O_3_ plays an important role in microplasma inactivation. Interestingly, O_3_ appears in the microplasma with N_2_ as carrier gas, and the concentration range is 56.5 ± 0.5 ppm. It shows that some active species in N_2_ microplasma ejected from the nozzle can react with O_2_ quickly in the air to generate O_3_. The related equation is as follows:


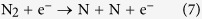



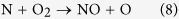



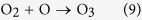


It suggests that the ionization product of O_2_ and N_2_ in the discharge area can react with O_2_ to generate the persistent inactivation material, O_3_. Besides, the degree of gas ionization and the oxygen concentration in the surrounding environment determine the ozone concentration.

## Mechanism analysis

### Inactivation effect analysis

**Figure 11:**
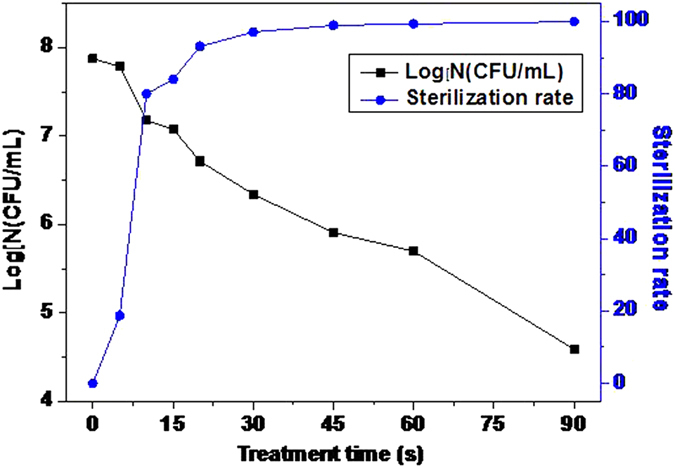
The inactivation effect of microplasma: the inactivation process appears first quick back slow trend.

Due to having changed the preparation method of dye bacterium, it is necessary to carry on the research of inactivation effect firstly. The results of logarithm concentration and inactivation rate of the live bacteria under different processing time are shown in Fig. 11.

The experimental results show that the process of microplasma jet inactivation is mainly divided into two stages of 0 to 30 s and 30 s −120 s. The former is the stage of rapid inactivation. The bacteria logarithmic reduction is 1.54 within 30 s, and the inactivation rate reaches 97.1%. The latter is the stage of slow inactivation. The bacteria logarithmic reduction is only 1.75 within 60 s, and it only inactivates 2.9% of the total number of bacteria actually. It can be known from the figure that with air as carrier gas, inactivation process appears first quick back slow trend.

## Analysis of intracellular content escaping

### Change of ion concentration

K and Mg element is the important components of bacteria cells. They exist in the bacterial cytoplasm mainly in the form of inorganic salt. In order to explore the changes of the permeability and rupture of cell membranes, the concentration changes of K^+^ and Mg^2+^ in supernatant of the samples were studied.

**Figure 12:**
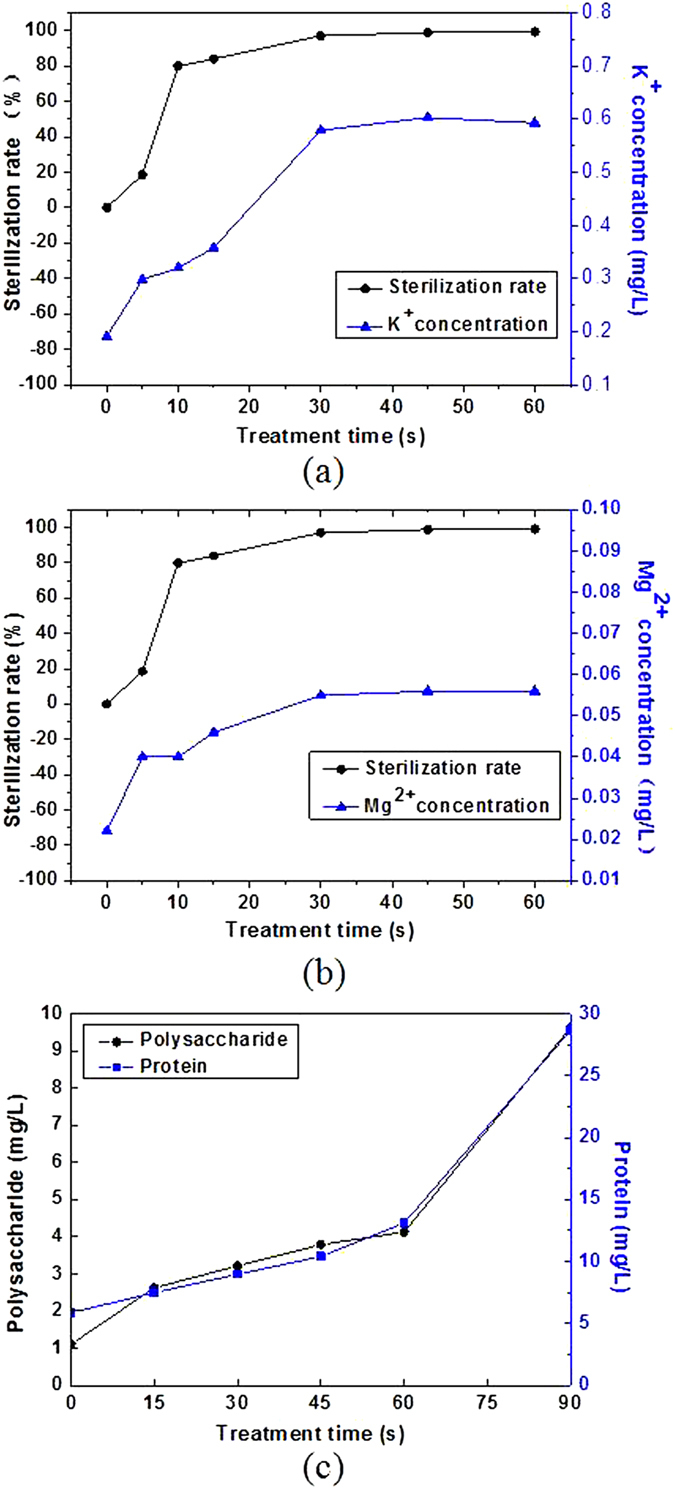
(**a**) The change trend of K^+^ concentration during the inactivation process: 0 to 30 s is the fastest rising stage of ion concentration; (**b**) The change trend of Mg^2+^ concentration during the inactivation process: 0 to 30 s is the fastest rising stage of ion concentration; (**c**) The changes of protein and polysaccharide in the supernatant: the concentrations of the polysaccharide and protein grow in the 90 s, and after 60 s, the concentrations both grow exponentially.

In the experiments, ICP-AES was used to detect the ion concentration in the supernatant. The concentration changes of K^+^ and Mg^2+^ are shown in Fig. 12(a,b).

From Fig. 12(a,b), 0 to 30 s is the fastest rising stage of ion concentration. In that stage do most bacteria inactivate, which proves that the selective permeability of cell membrane has failed. As for inactivation rate, the stage of fastest change is 5–10 s.

The reason for that case is as follows: cell membrane has certain protective effect^36^. The etching effect of microplasma jet can change the permeability of bacterial cell membrane, or even destroy the structure of cell membrane. When the selective permeability of cell membrane has failed, K^+^ and Mg^2+^ can escape. But if plasma does not continue to function, the structure of cell membrane will not be destroyed, and bacteria can also “revive”. That explains well the permeability of cell membrane changes in the first 5 s. It is due to that the effect of plasma and element concentration in the supernatant fluid rise rapidly. But the inactivation effect shows “delayed” due to the protective function of cell membrane. The structure of cell membrane is severely damaged through the further etching effect of plasma from 5 s to 10 s. And the failure of protection mechanism makes cell cracking and 60% of the bacteria be inactivated. In addition, the “delayed” phenomenon is more obvious for gram positive bacteria and spores which have stronger protection mechanism.

### Change of protein and polysaccharide

It has be learned that the cell membrane has protection mechanism. In order to further explore the etching effect of the microplasma jet on the cell membrane of *E. coli*, the changes of protein and polysaccharide in the supernatant were detected after inactivation treatment. Protein was detected by Folin-phenol method (also called Lowry method), and polysaccharide was detected by sulfuric acid-phenol method. The experimental results are shown in Fig. 12(c).

Figure 12(c) shows that the concentrations of the polysaccharide and protein grow in the 90 s. The growth is relatively stable during 0–60 s. But after 60 s, the concentrations both grow exponentially, making the concentration of protein and polysaccharide close to 30 mg/L and 10 mg/L, respectively.

The main structure of *E. coli* includes cell wall, cell membrane, cytoplasm and ribosomes. The major components of them are protein and polysaccharide. When microplasma imposes on the bacterial, the permeability of cell wall and cell membrane increases or they directly burst. It results in the cytoplasm and the ribosome escaping, which accounts for the rising concentration of polysaccharide and protein in 60 s. As can be seen from Fig. 11, almost all bacteria have been inactivated after 60 s. And compared with the content changes of K^+^ and Mg^2+^, it can be known that the structure of cell wall and cell membrane is destroyed as well and the cytoplasm has escaped largely. Furthermore, the fragmentization of cell wall and cell membrane will appear with continuous treatment by microplasma. That leads to the sharp increase of the concentrations of protein and polysaccharide during the 60 s–90 s. So it can be concluded that the microplasma jet can not only inactivates microorganisms but also remove the body like other plasma^37^.

### Change of soluble organic matter

**Figure 13:**
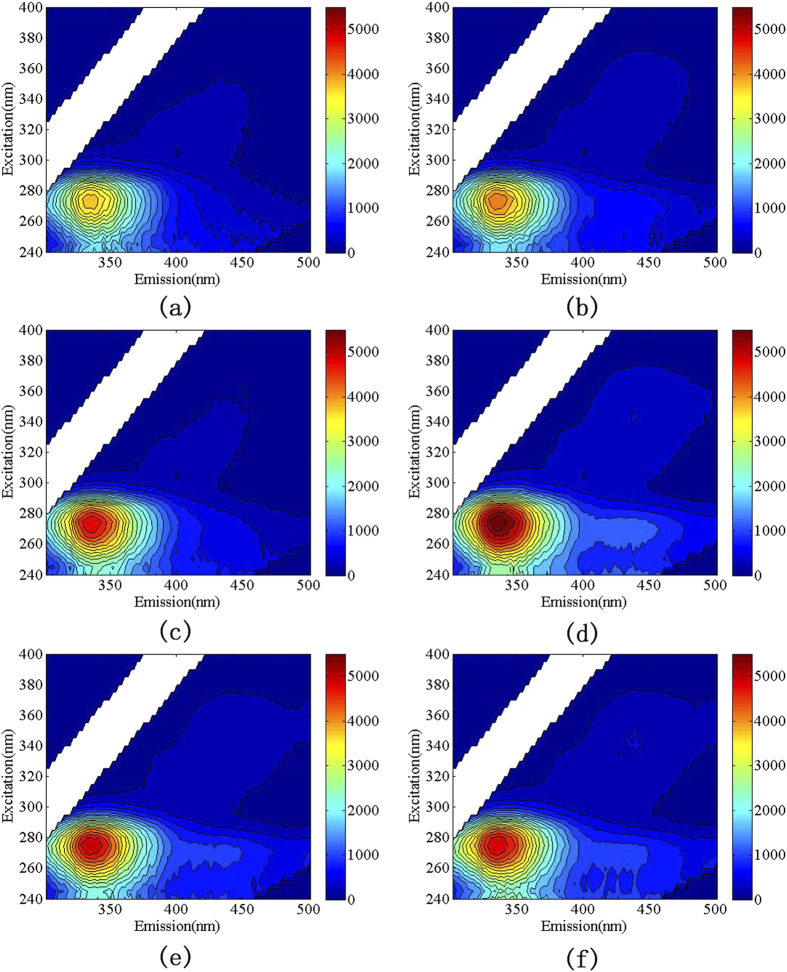
The results of 3D-EEM treated with different times (a-0 s, b-10 s, c-15 s, d- 30 s, e-45 s, f-60 s).

The content change of soluble organic matter was studied by 3D-EEM with high sensitivity and selectivity. The result is shown in Fig. 13.

As can be seen from the six pictures of Fig. 13, the peaks of fluorescence intensity are located in the same position, namely the Ex/Em being 265–280/325–345. It is because the samples treated by microplasma is from the same kind of *E. coli*. Within the first 30 s, the peak height of fluorescence intensity increases with the extension of treatment time. It suggests that intension gradually strengthens. Compared to the inactivation rate of Fig. 13, it can be thought that during this period, the plasma jet leads to the cell membrane rupture and cytoplasm escaping. That causes the concentration of soluble organic matter in bacterium solution increase. After 30 s, the fluorescence intensity gradually reduces with the extension of treatment time. It is mainly on account of the oxidation of soluble organic matter by microplasma jet^38^. And the result of Fig. 12(c) indicates that the concentration of organic matter continuously growth during the 90 s, especially during the 60 s–90 s. That may be due to that cell wall and cell membrane of the bacterial is in continuing fragmentation. And fragmentation of cell wall and cell membrane is not necessarily soluble organic matter. That leads to that the concentration of protein and polysaccharide increases but the soluble organic matter reduces after 30 s.

That means microplasma jet can not only inactivate the microbes but oxygenize and decompose the residual organic matter after the death of microbial. It has the function of cleaning the surfaces contaminated by organic matter^22^,^37^.

### Change of cell morphology

The experiments of K^+^ and Mg^2+^ and proteins and polysaccharides have proved the microplasma can lead to the cell membrane rupture, resulting in the death from the side. In this part SEM was used to study the change of cell morphology of *E. coli* in the process of microplasma treatment^39^.

**Figure 14:**
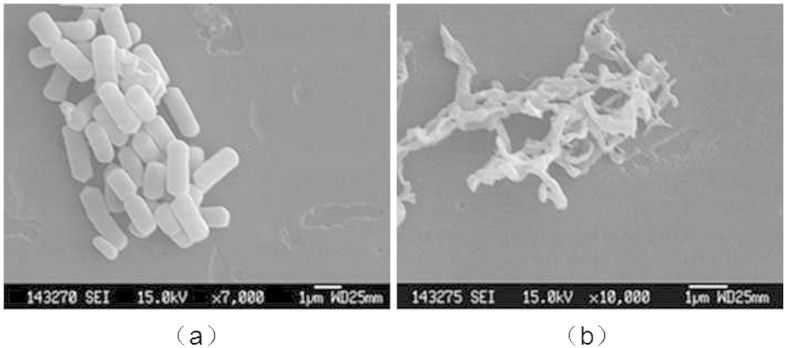
The SEM images before and after treatment: Bacterial cells before treatment presented full status, complete morphology, smooth surface, showing regular rhabditiform; cell morphology occurred serious deformation, appearing dry and imperfect after treatment for 60 s.

**Figure 15:**
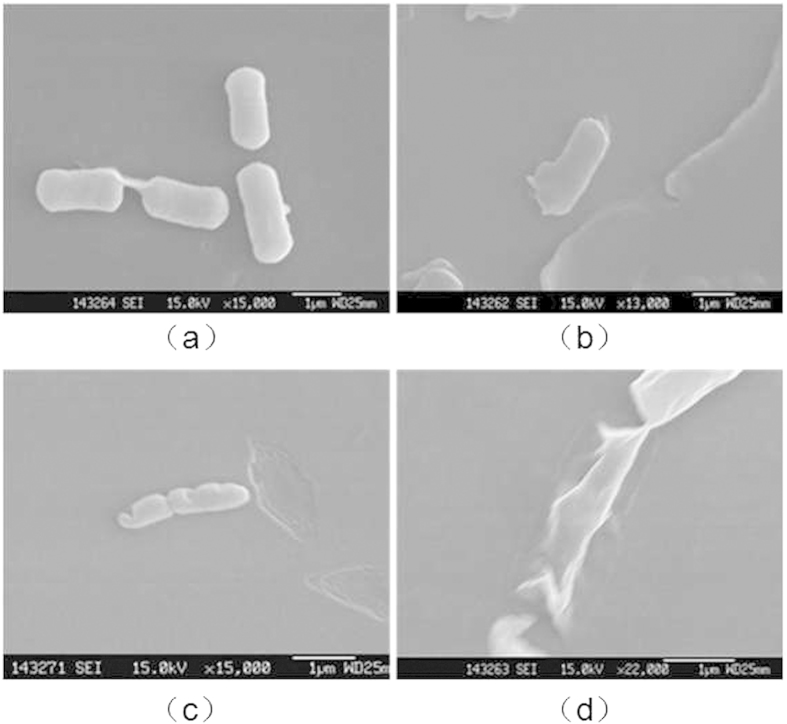
The SEM images of different treatment times: (**a**) bacterial cells before treatment presented complete morphology and smooth surface, showing regular rhabditiform; (**b**) cell membranes occurred only rupture slightly and it is no longer the original regular rhabditiform; (**c**) cells occurred much rupture in the cell membrane, cell morphology was severely incomplete; (**d**) bacterial cytoplasm completely outflowed, and cell morphology occurred serious deformation.

It can be found from the SEM images before and after treatment that cell morphology of *E. coli* changes a lot. Bacterial cells before treatment presented full status, complete morphology, smooth surface, showing regular rhabditiform (as shown in Fig. 14(a). But after treatment for 60 s, the cell morphology occurred serious deformation, and single cells were elongated. In addition, they appeared dry and imperfect (as shown in Fig. 14(b). That is due to that the microplasma treatment led to cell membrane rupture and cytoplasm escaping. It proved again from the angle of morphology that the inactivated mechanism of *E. coli* in the process of microplasma treatment is primarily therupture of cell membrane. In order to learn the detailed changes of cell morphology during the processing of 60 s, the samples treated for 0 s, 15 s, 30 s, and 60 s were observed by electron microscopy. The more clear SEM images of single cell were obtained with greater magnification as shown in Fig. 15.

It can be found from the SEM that the bacterial cells before treatment presented complete morphology and smooth surface, showing regular rhabditiform (as shown in Fig. 15(a). After the treatment, the microplasma had etched on the cell wall and cell membrane of bacterial. And there were mainly three different etching conditions: cell membranes occurred only rupture slightly and it is no longer the original regular rhabditiform (as shown in Fig. 15(b); cells occurred much rupture in the cell membrane, cell morphology was severely incomplete, and the size was also smaller due to the cytoplasm massively outflowed (as shown in Fig. 15(c); bacterial cytoplasm completely outflowed, and cell morphology occurred serious deformation, only remaining the dry pieces (as shown in Fig. 15(d). That explains the phenomenon that the concentrations of protein and polysaccharide of bacterium solution dramatically increase in Fig. 12(c).

Those three different etching conditions can also be regarded as three different stages of *E. coli* treated by microplasma. And the cell morphology of *E. coli* changed step by step as the etching effect changed. It proves that the etching effect of microplasma jet resulted in the cell membrane rupture and bacteria death as well.

## Conclusions

In this paper, with *E. coli* as the target microorganisms, the qualitative and quantitative researches were conducted to study the inactivation effect of microplasma jet. The main conclusions are as follows:

The discharge of microplasma jet is extremely unstable, but both periodically change. And the discharge shows greater instability with higher gas flow rate. For air microplasma, with the increase of air flow rate, inactivation effect improves first and then weakens; with the increase of inactivation distance, the inactivation effect of microplasma weakened gradually; the comparison of inactivation effect with different carrier gases shows that oxygen microplasma jet has the strongest inactivation ability, followed by air, nitrogen and argon.

Heating effect and ultraviolet radiation in air microplasma jet basically do not possess inactivation ability alone. Within the first 30 s, the vast majority of bacteria have been dead, but the temperature on the bacterial carrier is still less than 43 °C. Though charged particles have certain ability of inactivation, there is no obvious change compared with the control group. That means charged particle is not the main factor of microplasma inactivation but the neutral active species is.

The active species in air microplasma are mainly generated by the ionization of oxygen and nitrogen. Taking ozone as an example, with the increasing air flow, ozone concentration increases first and then decreases. The change trend is highly consistent with the inactivation effect. That proves that the concentration of the active species determines the strength of the inactivation effect.

Microplasma jet has etching effects on cell membranes of *E. coli.* With the extension of treatment time, the cell membrane permeability of bacterial begins to change. And finally cell membrane cracking occurs, leading to various ions, proteins and polysaccharides escaping. And cell morphology is transformed from originally full and smooth to irregular and dry.

## Additional Information

**How to cite this article**: Du, C.M. *et al.* Qualitation and Quantitation on Microplasma Jet for Bacteria Inactivation. *Sci. Rep.* **6**, 18838; doi: 10.1038/srep18838 (2016).
